# Lateral semi-circular canal asymmetry in females with idiopathic scoliosis

**DOI:** 10.1371/journal.pone.0232417

**Published:** 2020-04-29

**Authors:** Patrick M. Carry, Victoria R. Duke, Christopher J. Brazell, Nicholas Stence, Melissa Scholes, Dominique L. Rousie, Nancy Hadley Miller

**Affiliations:** 1 Department of Orthopaedic Surgery, Musculoskeletal Research Center, Children’s Hospital Colorado, Aurora, Colorado, United States of America; 2 Department of Radiology, Children’s Hospital Colorado, Aurora, Colorado, United States of America; 3 Department of Otolaryngology, Children’s Hospital Colorado, Aurora, Colorado, United States of America; 4 La Fondation Cotrel, Institut de France, Paris, France; Ohio State University, UNITED STATES

## Abstract

**Purpose:**

Adolescent idiopathic scoliosis (AIS) is a three-dimensional spinal structural deformity that occurs in otherwise normal individuals. Although curve progression and severity vary amongst individuals, AIS can lead to significant cosmetic and functional deformity. AIS etiology has been determined to be genetic, however, exact genetic and biological processes underlying this disorder remain unknown. Vestibular structure and function have potentially been related to the etiopathogenesis of AIS. Here, we aimed to characterize the anatomy of the semicircular canals (SCC) within the vestibular system through a novel approach utilizing T2-weighted magnetic resonance images (MRI).

**Methods:**

Three dimensional, MRI-based models of the SCCs were generated from AIS subjects (n = 20) and healthy control subjects (n = 19). Linear mixed models were used to compare SCC morphological measurements in the two groups. We compared side-to-side differences in the SCC measurements between groups (group*side interaction).

**Results:**

Side-to-side differences in the lateral SCC were different between the two groups [false discovery rate adjusted p-value: 0.0107]. Orientation of right versus left lateral SCC was significantly different in the AIS group compared to the control group [mean side-to-side difference: -4.1°, 95% CI: -6.4° to -1.7°]. Overall, among subjects in the AIS group, the left lateral SCC tended to be oriented in a more horizontal position than subjects in the control group.

**Significance:**

Asymmetry within the SCCs of the vestibular system of individuals with AIS potentially results in abnormal efferent activity to postural muscles. Consequences of this muscular activity during periods of rapid growth, which often coincides with AIS onset and progression, warrant consideration.

## Introduction

Late onset or adolescent idiopathic scoliosis (AIS) is a three-dimensional spine abnormality present in 1–3% of children ages 10 to 16 years old [1–4]. Because the etiopathogenesis of AIS is unknown [5], interventions are directed at the anatomic structural deformity rather than the underlying cause of deformity. Recent evidence suggests the vestibular system may play a role in the etiology of AIS [6–9], due to its impact on the hypothalamus, cerebellum, and vestibulospinal pathway [10].

The vestibular system is comprised of otolith organs and three orthogonal semicircular canals (SCCs) [11]. Each semi-circular canal works in tandem with its partner on the contralateral side. Angular accelerations result in hair cell deflections within the SCCs that provide afferent signals regarding the direction and intensity of the movement [12, 13]. Together, these signals aid in balance and postural control. Angular acceleration sensitivity is directly correlated with canal morphology [14], suggesting that any structural abnormality may cause downstream effects including impaired balance and postural muscle activity. Since the SCCs have a fixed size and shape at birth [10, 15, 16], abnormalities may play an early causative or contributory role in the pathogenesis of AIS through activation of paraspinous muscles responsible for trunk support [3]. Previous studies have identified vestibular morphological anomalies among individuals with AIS when compared to normal controls [10, 17]. However, controversy exists regarding the role of SCC canal morphology in AIS [18, 19].

We aimed to establish a novel approach to imaging the semicircular canals to assess the association between anatomical variation in SCCs and AIS. We tested whether side-to-side differences in SCC geometry are exaggerated in patients with AIS relative to control subjects.

## Materials and methods

### Patients and methods

Adolescent females undergoing treatment for moderate to severe AIS at a single institution (n = 20) were recruited for the AIS study group. The location, type, and direction (side of curve convexity) of the curves was recorded for all subjects (see Table 1). Adolescent and young adult females (n = 19) without scoliosis were recruited as a control group. To see the full data set of demographics and data dictionary used, please refer to S1 Table and S2 Table. The Colorado Multiple Institution Review Board (COMIRB) has reviewed and approved this study. The COMIRB study number is 09–0706. The original "certificate of approval" was provided on 09/24/2009. Consent and assent (when needed) was given in written fashion with COMIRB approved consent forms.

**Table 1:** Scoliosis curve patterns.

Patient	Curve Type	Cobb Angle†
1	R. Thoracic	47°
2	R. Thoracic	39°
3	R. Thoracic	56°
4	Biphasic	42°/46°
5	Biphasic	50°/50°
6	R. Lumbar	50°
7	R. Thoracic	42°
8	Biphasic	34°/35°
9	Biphasic	54°/52°
10	Biphasic	26°/29°
11	R. Thoracic	52°
12	R. Thoracic	40°
13	R. Thoracic	42°
14	Biphasic	42°/38°
15	Biphasic	58°/54°
16	R. Thoracic	56°
17	Biphasic	52°/48°
18	R. Thoracic	40°
19	L. Lumbar	60°
20	R. Thoracic	70°

R = right convexity

L = left convexity

Biphasic or double major = R thoracic / L thoracolumbar curves of similar magnitude

†Radiographs obtained at most recent visit following skeletal maturity or radiograph prior to surgical intervention

### Imaging protocol

A 1.5T MRI (Siemens) was used to obtain T2-weighted images of the vestibular system under the following conditions: Avonto True FISP sequence, repetition time of 4.93 ms, echo time of 2.16 ms, flip angle of 65˚, percent phase field of view (FOV) of 75, 1 mm slice thickness, matrix of 256 x 192, and 1 excitation. This process yielded anisotropic images with voxel size 0.98 x 0.98 x 1.0 mm^3^.

All Digital Imaging and Communications in Medicine (DICOM) data were imported into 3D-Slicer [20] version 4.7.0. The original voxel size was reduced to 0.28 x 0.28 x 0.28 mm^3^ following b-spline interpolation. Salient edges were then smoothed and defined using a gradient diffusion filter. In order to ensure consistent measurements of vestibular systems in relation to anatomical landmarks, images were then reoriented according to the anterior (AC) and posterior commissure (PC) [21].

### Vestibular system segmentation

The SCCs were segmented from the preprocessed image using a process similar to the methodology developed by Shi et al [13]. A general, cubic region of interest (ROI) was first extracted for each left and right vestibular system from the input T2 weighted image of the whole head to increase processing speed. The surface of the vestibular system was extracted using the competitive region growing algorithm *GrowCut* to select like pixels and create a 3D triangular mesh from systematically selected initial conditions [22]. Because segmentation can be largely dependent on initial conditions, the first generated surface mesh was then used as the initial condition to generate a second triangular mesh that was used in subsequent analyses.

### SCC measurements

**Fig 1:**
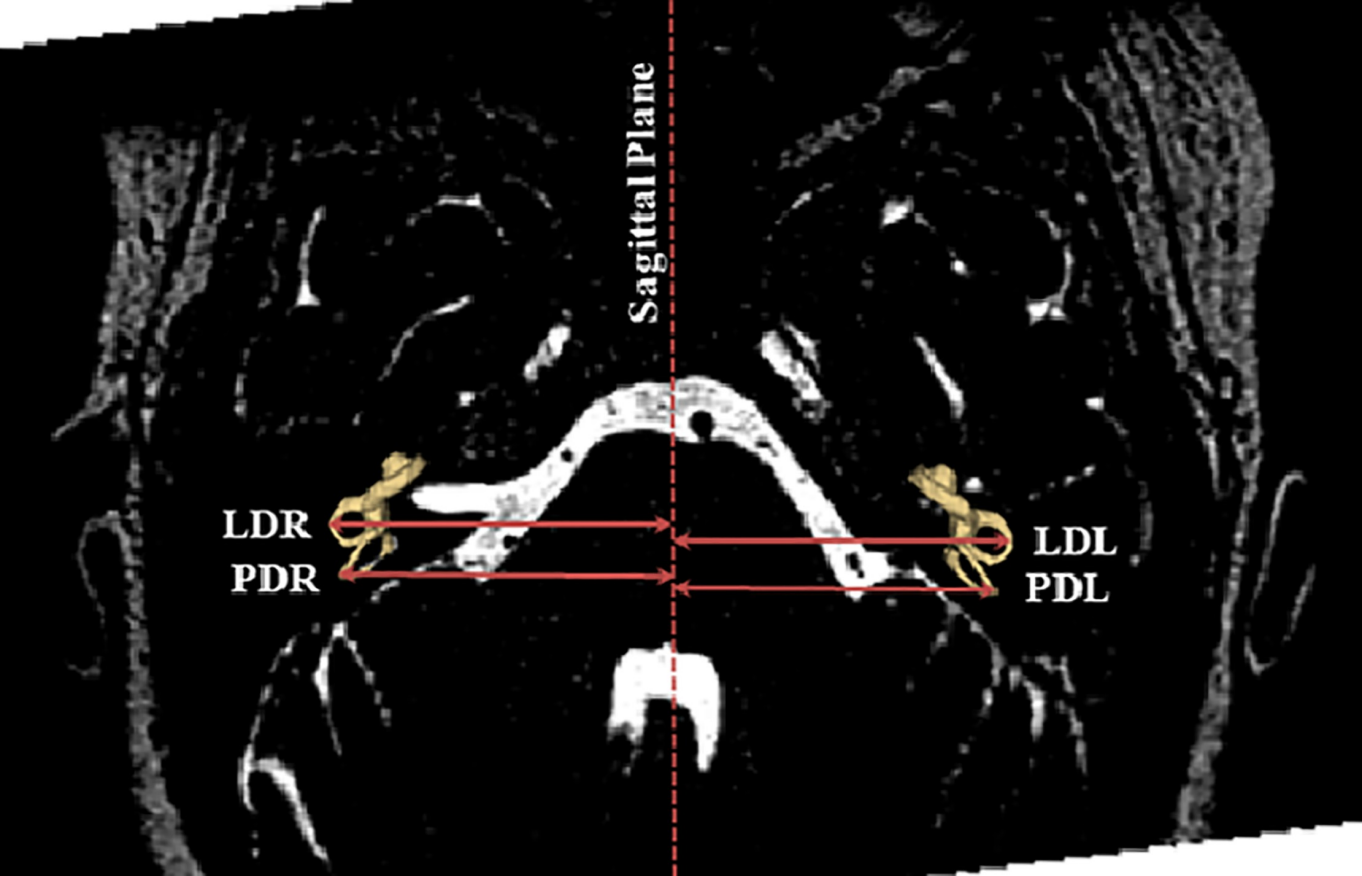
SCC distance measurements. Measurements of the horizontal distance from the lateral and posterior canals to the sagittal plane midline. LDR = lateral canal distance on the right, LDL = lateral canal distance on the left, PDR = posterior canal distance on the right, PDL = posterior canal distance left.

**Fig 2:**
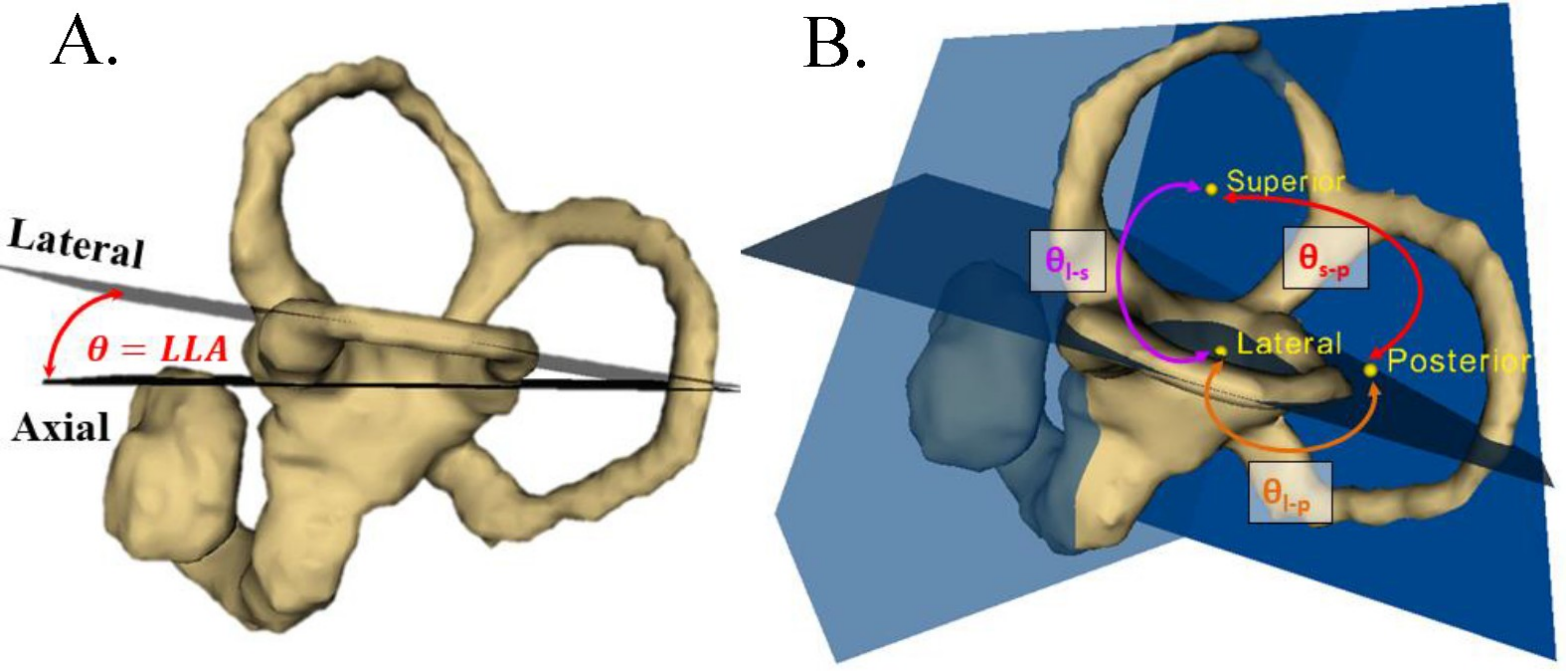
SCC angle measurements. (A) Angle (θ) between the axial and best-fit left lateral canal planes (LLA) on the reconstructed study surface. (B) Angles between each canal best-fit plane: *θ_l−s_* = angle between lateral and superior SCCs, *θ_l−p_* = angle between lateral and posterior SCCs, *θ_s−p_* = angle between superior and posterior SCCs.

We measured the horizontal distance from the lateral and posterior SCCs to the anatomical midline (defined as the line connecting the AC and PC). Accordingly, the surface mesh was superimposed on the reoriented whole head MRIs. The horizontal distance in the axial plane of the lateral and posterior canals was measured from the anatomic midline to the lateral most aspect of the lateral and posterior canals, respectively (Fig 1). The lateral, superior, and posterior SCCs were then separated from the overall vestibular system model. SCC endpoints were defined as the vestibule and the bifurcation of the common crus. This was determined by reviewing changes in cross-sectional area, allowing the operator to identify one common intersection point between the superior (anterior) and posterior canals. An approximate centerline for each canal was manually defined to divide the canal into cross-sectional slices spaced by 0.25mm using the *IGT Volume Reslice Driver in Slicer* [23]. The canal centroid was then defined using spline interpolation between the center of mass calculated for each division. Cross-sectional area measurements were taken at each of the divisions resulting from the *IGT Volume Reslice Driver*. Best-fit planes were determined following the conversion of each surface model to a volumetric cloud. Canal planes were fit relative to the inverse of the cross-sectional area squared as this technique has been suggested to best represent the functional plane [14, 24]. These planes were then utilized to calculate the angle between the canal planes as well as their angles compared to coordinate reference planes using the dot product of the two-plane normal vector (Fig 2 and S1 Fig for more information). For the full data set of measurements, please refer to S3 Table.

### Statistical methods

We compared side-to-side differences (right SCC measure minus the left SCC measure) in the two groups using linear mixed models. We tested the null hypothesis of no difference in side-to-side differences across groups (group*side interaction). The unstructured covariance structure was used to account for repeated measures (R and L side). The following parameters were compared: (1) distance from each lateral SCC to the midline, (2) distance from each posterior SCC to the midline, (3) inclination of each lateral SCC in the sagittal plane, (4) minimum cross-sectional area of each superior SCC, (5) minimum cross-sectional area of each posterior SCC, (6) minimum cross-sectional area of each lateral SCC, (7) angle between each superior SCC and each lateral SCC best-fit-planes, (8) angle between each superior SCC and posterior SCC best-fit-planes, and (9) angle between each posterior SCC and lateral SCC best-fit-planes. Due to the large number of statistical comparisons, the Benjamini-Liu [25] method was used to control the false discovery rate (FDR). Statistical significance was defined as an FDR corrected p value <0.05.

## Results

The average age at the MRI visit was higher in the control group (22.0 yrs. ±7.8) relative to the AIS group (14.6 yrs. ±1.9). The majority of subjects in the AIS (18/20, 90%) and control (14/19, 74%) groups were non-Hispanic white. Curve patterns in the AIS group included primary thoracic (10/20, 50%), biphasic or double major (8/20, 40%), and lumbar (2/20, 10%). All primary thoracic and biphasic curves had a thoracic component with convexity, in the coronal plane, that was oriented to the right. The average Cobb angle was 48.4° (±10.0) among individuals with a single primary thoracic curve, 46.1° (±10.2) among individuals with biphasic curves, and 55.0° (±7.1) among individuals with a single primary lumbar curve.

Side-to-side differences in the lateral canal angle (R minus L) were significantly different between the AIS and control groups (FDR adjusted p value <0.05, Table 2). The average difference in the lateral SCC angle between R and L sides was 3.6° [95% CI: 1.94 to 5.2°] in the AIS group compared to -0.5° [95% CI: -2.2 to 1.2°] in the control group. Overall, the side-to-side difference in the lateral SCC angle was 4.1° [95% CI: 1.7 to 6.4°] higher in the AIS group relative to the control group. The side-to-side differences in the other SCC parameters were not significantly different (FDR adjusted p value >0.05) between the AIS and control groups (Table 2).

**Table 2:** SCC canal measurements by group and side.

	IS Group		Control Group			
	Left	Right	Left	Right	P value*	FDR Adjusted P value**
	Mean (Stdev)	Mean (Stdev)	Mean (Stdev)	Mean Stdev)		
Angle between the superior and lateral SCC best-fit planes	88.72 (6.85)	89.72 (4.52)	92.06 (5.90)	92.04 (6.97)	0.6573	0.6230
Angle between the superior and posterior SCC best-fit planes	87.47 (3.44)	87.00 (3.73)	88.02 (4.54)	86.74 (5.23)	0.6669	0.6230
Inclination of the lateral SCC in the sagittal plane	1.46 (3.42)	5.03 (2.15)	5.21 (3.03)	4.72 (3.88)	0.0012	0.0107
Distance from midline to lateral SCC	42.74 (2.12)	43.20 (1.89)	44.12 (1.89)	43.86 (1.94)	0.3652	0.6230
Minimum cross-sectional area: superior SCC	1.27 (0.74)	1.20 (0.68)	1.31 (0.72)	1.21 (0.51)	0.8941	0.6230
Minimum cross-sectional area: lateral SCC	1.50 (0.85)	1.51 (0.80)	1.26 (0.61)	1.41 (0.64)	0.4356	0.6230
Minimum cross-sectional area: posterior SCC	1.87 (0.97)	1.43 (0.64)	1.30 (0.55)	1.39 (0.47)	0.0604	0.3226
Angle between posterior and lateral SCC best-fit planes	91.07 (4.70)	89.30 (4.81)	85.71 (4.68)	87.65 (5.28)	0.0548	0.3226
Distance from midline to posterior SCC	40.8 (2.11)	41.20 (2.16)	41.86 (2.31)	41.89 (2.13)	0.5124	0.6230

*Nominal p value

**FDR corrected p value (Benjamini-Liu method [25]) representing group*side interaction, Stdev = standard deviation, SCC = semi-circular canals

To assess the heterogeneity in SCC lateral angle measurements on the basis of curve type, we also summarized lateral SCC angle measurements among subjects with a primary thoracic pattern, biphasic curve pattern, or a primary lumbar curve pattern (Table 3). Relative to the control group, right sided lateral angle measurements were consistently increased (positive side-to-side difference) among subjects with a primary thoracic curve pattern, biphasic curve pattern, as well as subjects with a primary lumbar curve pattern (Table 3).

**Table 3:** Measurements by curve type.

Curve Type	n	Mean Difference (R—L)	Standard Deviation
Control	19	-0.48	3.25
Biphasic	8	4.36	3.95
R. Thoracic	10	2.87	4.15
R. Lumbar	1	6.63	n/a
L. Lumbar	1	1.13	n/a

## Discussion

The etiology of AIS and subsequent curve progression remains poorly understood. An association between vestibular system abnormalities and AIS has been reported in current literature [6, 10, 13, 26]. Using three-dimensional MRI based models of SCC anatomy, we compared measurements of SCC morphology in females with AIS relative to female controls. Analysis of side-to-side differences in SCC measurements revealed significant asymmetry in the orientation of the lateral SCC in subjects with AIS relative to control subjects. The left lateral SCC tended to be orientated in a more horizontal position among subjects in the AIS group compared to the control group. This may have important implications for the potential relationship between vestibular SCC anatomy and the etiopathogenesis of AIS.

The results of the present study are consistent with several studies that have identified lateral canal abnormalities among patients with AIS. Shi et al. found the distance between the centers of the left lateral and superior canal were significantly smaller in 20 AIS patients with right thoracic curvatures compared to 20 controls [13]. After evaluating the semicircular canals in 18 AIS patients with right and/or left curvatures, Hitier and colleagues found that the left lateral semicircular canals were more vertical and further from the midline compared to 9 controls [10]. While these findings may seem discordant relative to our findings, it should be noted that Hitier utilized 2D measurements with manual measurements as compared to our MRI derived measurements that evaluated the angle of the best-fit lateral planes in three dimensional space, and thus may provide a more functional assessment of the lateral SCC plane. Three additional MRI studies analyzing the semicircular canals with 3D reconstruction have highlighted lateral canal abnormalities present on both sides in AIS patients [26–28]. These include an abnormal “fluid bridge” between the lateral and posterior canals [26], and longer, thinner canals [27, 28].

Studies of computational fluid-flow within idealized SCCs have suggested that regions of small cross-sectional area play a fundamental role in the movement of endolymph through the vestibular system, causing them to dominate biomechanical sensitivity [11, 14]. Complex MRI analysis of 15 AIS patients compared to 12 controls revealed thinner lateral canals with significant shape differences [28]. These findings were confirmed by Xin and colleagues who observed longer, thinner canals in 11 female girls with right thoracic AIS compared to 11 healthy controls [27]. We compared the minimum cross-sectional area of each canal in our study population but observed no difference in the cross-sectional area measurements in the AIS group relative to the control group.

### Theoretical model

Several hypotheses have been proposed to explain the connection between asymmetric SCC anatomical variation and scoliosis. Central to these hypotheses is the assumption that the SCC structure or orientation affects balance and consequently, postural muscle activity. Afferent signals from the vestibular system are interpreted by higher brain centers to maintain postural support and balance [12]. Based on latency responses observed in mammalian neurons activated by the vestibular system, it has been suggested that vestibular afferents form reflex arcs with antigravity trunk musculature [29–34]. Although labyrinth output is bilateral, excitatory signals from each labyrinth influence contralateral postural and lower limb muscle activity in the coronal plane [34]. Therefore, it is conceivable that asymmetrical or unilateral hypofunction in vestibular afferent activity could result in unequal efferent output to the postural muscle motor neuron pools of the trunk. This could potentially explain the overrepresentation of both left-sided SCC abnormalities[10, 13] and of right sided thoracic curves [35] among patients with AIS. However, this remains purely speculative and thus, the consequences of SCC variation on muscular activity during periods of rapid growth, such as puberty, which often coincides with the AIS onset and progression, warrants consideration.

Abnormal posture and balance in patients with AIS is well documented [19, 28, 36–38]. Following vestibular injury, balance compensation can occur from higher brain centers. However, only static control is regained. Externally imposed vestibular stimulations after unilateral vestibular loss causes impaired responses that do not recover [39]. Therefore, while unilateral canal loss has been reported to be compensated for [3], dynamic functioning is not recovered [39]. This may explain why patients with AIS can perform normally under static conditions, but struggle with sensory challenged positions [37]. It is important to note that body verticality and balance are controlled by visual, somatosensory, and vestibular inputs [40], with proprioception playing the largest role in the control of upright body posture and orientation [41]. Because normal adolescents have a transient period of proprioceptive neglect compared to adults [42], it may be possible that semicircular canal abnormalities present at birth have a magnified effect during adolescence.

Animal models simulating conditions of exaggerated unilateral vestibular hypofunction through selective lesions to vestibular system anatomy have also provided evidence to support the role of asymmetric vestibular system activity in the development of abnormal spinal curvature. Selective lesions of the vestibular nucleus in a rat model produced scoliosis with an average Cobb angle of 21° in 15% of the surviving rats after eight weeks [43]. Selective hemilabyrinthectomy in guinea pigs produced a postural disorder and a lateral curvature of the vertebral column that strongly resembled scoliosis [7, 44]. The convexity of the curvature in the guinea pigs was consistently orientated towards the intact or unaffected labyrinth. This coincides with Lambert et. al who reported that unilateral destruction of the vestibular system in a frog model caused a spinal curvature with a convexity that was oriented toward the intact or unaffected vestibular system [8]. In contrast to other animal studies in which the scoliotic deformity tended to be temporary, the scoliotic deformity in the frog model was permanent. The authors suggested the lack of forelimb proprioceptive feedback in the aquatic environment may prevent compensation from forelimb proprioceptive feedback, a hypothesis that may be relevant to the bipedal nature of human ambulation that similarly lacks forelimb proprioceptive feedback.

Among observational studies in adolescent and adult populations, support for the hypothetical role of vestibular dysfunction in AIS has been inconsistent. A recent systematic review of level III-IV evidence concluded there is a causative role of the vestibular system in scoliosis [3], however, another systemic review concluded there is not enough research to support a consistent association between unilateral, isolated, vestibular dysfunction and AIS [1]. Additional targeted work is needed to understand the prevalence of SCC morphological asymmetries in individuals with AIS and the potential functional consequences of these abnormalities on the development and progression of spine deformities.

### Limitations

There are limitations to our study design. While there is discordant information surrounding functional testing of AIS patients, functional tests in the current study group would have been beneficial. In particular, otolithic tests (Curthoys test) in patients with abnormal lateral canals, represent the next logical experiment necessary to understand the association between SCC structural variation and scoliosis. Secondarily, the study population was limited to female subjects based on higher prevalence of more severe AIS in females compared to males. The results of the current study are not generalizable to AIS in male subjects. Subjects in the control group were older than subjects in the AIS group. This should not have an impact on our findings as the SCCs are fully developed at birth [45], and do not change orientation following embryogenesis [10, 46]. Confounding is also a major concern in case-control studies. By focusing on side-to-side differences in SCC morphology, the current study design avoids usual concerns regarding confounding as each subject acts as his/her own control subject. This method is consistent with methodology used by Hitier et al (2015) [10]. Lastly, the utricle and/or saccule also have potential relevance to idiopathic scoliosis based on their role in body and head posture. We did not review the utricle and/or saccule because the membranous utricle is impossible to visualize ‘in-vivo’ due to the limited resolution of the MRI. Finally, modelling approach in current study used some subjective operator input to develop the SCC models. Cortes Dominguez et al [47] describes a novel modelling approach that may decrease the subjective nature of the modeling strategy, resulting in a more accurate representation of underlying SCC. In our study, all images were de-identified and the assessor was blinded to case status. As a result, although we acknowledge that the inherent subjectivity in our methods may result in some measurement error, we expect this bias to be non-differential.

## Conclusions

We developed a method for analyzing the semicircular canals in three dimensions. Our results confirm previous studies [10, 13] that have observed an overrepresentation of morphological asymmetries within the left lateral SCC among patients with AIS. Compared to the right side, AIS patients have a more horizontally oriented left lateral semicircular canal than control subjects. These results potentially support the hypothesis that vestibular abnormalities may represent a latent pathology that is related to or contributes to the onset of AIS. The current study is observational in nature and thus, it is unclear whether anatomical asymmetries are causal or whether the SCC asymmetries and severe curve patterns are related to a common, upstream genetic variant. Additional work is needed to fully understand the fluid flow and functional balance consequences that may result from SCC geometrical asymmetries as well as the potential genetic origins of these abnormalities.

## Supporting information

S1 FigDetermination and utilization of best-fit planes for SCC angle.Best-fit planes were determined following the conversion of each surface model to a volumetric cloud of *m* data points according to the minimization of the following total squared error sum, representing the squared distance of point *x_i_* from the plane *ax+by+cz+d* = 0: $f(a,b,c,d)=\sum_{i=1}^{m}{\left|w_{i}*\overset{⃑}{n}∙(\overset{⃑}{x_{i}}-\overset{⃑}{c_{i}})\right|}^{2}$. Where $\overset{⃑}{n}$ is the plane normal vector, $\overset{⃑}{c}$ is the centroid path, $\overset{⃑}{x_{i}}$ is a vector containing the three-dimensional point taken from the volumetric model, and weight *w_i_* is proportional to the inverse, squared cross-sectional area of the nearest canal division: $w_{i}=\frac{1}{{A_{x_{i}}}^{2}}$. Canal planes were fit relative to the inverse of the cross-sectional area squared as this technique has been suggested to best represent the functional plane. [14, 24] Previous studies have used general measures of canal cross-sectional area to calculate canal planes. We implemented weights specific to the geometry of each patients’ canal to better reflect the functional maximal response plane, defined as the plane that exhibits maximal response when the three-canal system is rotated about that plane. These planes were then utilized to calculate the angle between the canal planes as well as their angles compared to coordinate reference planes using the dot product of the two plane normal vectors: $\theta ={cos}^{-1}(\overset{⃑}{n_{1}}∙\overset{⃑}{n_{2}})$.(TIF)

S1 Table Full data demographics. (XLSX)

S2 Table Full data measurements. (XLSX)

S3 Table Data dictionary. (XLSX)
