# Supplementary figures and images for: Lateral semi-circular canal asymmetry in females with idiopathic scoliosis
Source: PLoS One. 2020 Apr 29;15(4):e0232417. doi: 10.1371/journal.pone.0232417 (PMC7190182; doi:10.1371/journal.pone.0232417)

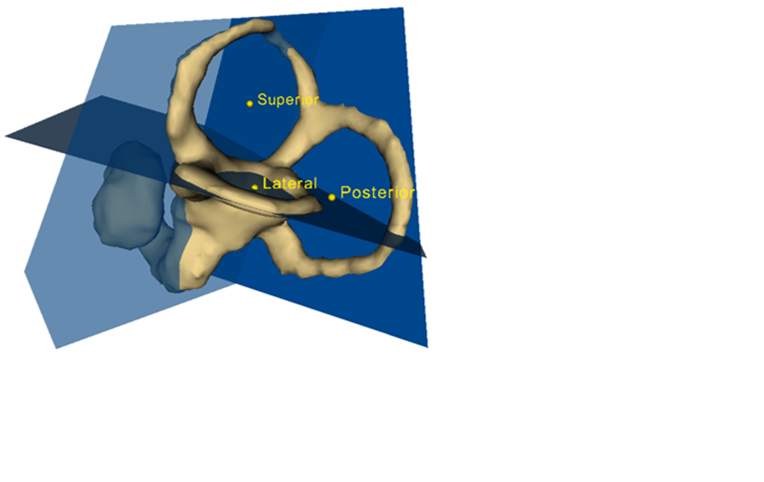

Supplement: S1 Fig — Best-fit planes were determined following the conversion of each surface model to a volumetric cloud of m data points according to the minimization of the following total squared error sum, representing the squared distance of point xi from the plane ax+by+cz+d = 0: f(a,b,c,d)=∑i=1m|wi*n⃑∙(xi⃑−ci⃑)|2. Where n⃑ is the plane normal vector, c⃑ is the centroid path, xi⃑ is a vector containing the three-dimensional point taken from the volumetric model, and weight wi is proportional to the inverse, squared cross-sectional area of the nearest canal division: wi=1Axi2. Canal planes were fit relative to the inverse of the cross-sectional area squared as this technique has been suggested to best represent the functional plane. [14, 24] Previous studies have used general measures of canal cross-sectional area to calculate canal planes. We implemented weights specific to the geometry of each patients’ canal to better reflect the functional maximal response plane, defined as the plane that exhibits maximal response when the three-canal system is rotated about that plane. These planes were then utilized to calculate the angle between the canal planes as well as their angles compared to coordinate reference planes using the dot product of the two plane normal vectors: θ=cos−1(n1⃑∙n2⃑). (TIF) [file pone.0232417.s001.tif]
